# Prolonged Invasive Mechanical Ventilation in a Tertiary Respiratory ICU: Outcomes, Challenges, and Future Directions

**DOI:** 10.7759/cureus.109455

**Published:** 2026-05-22

**Authors:** Rahul Tyagi, Gaurav Mishra, Manu Chopra, Prerna G, Siddhartha C, Shemsya Shajahan, Bharti Kushwaha

**Affiliations:** 1 Pulmonary, Critical Care, and Sleep Medicine, Army Institute of Cardiothoracic Sciences, Armed Forces Medical College, Pune, IND; 2 Respiratory Medicine, Armed Forces Medical College, Pune, IND; 3 Pulmonology, Armed Forces Medical College, Pune, IND

**Keywords:** acute hypoxemic respiratory failure, multidrug-resistant infections, prolonged mechanical ventilation, respiratory intensive care unit, tracheostomy, ventilator-associated pneumonia, weaning

## Abstract

Background

Prolonged invasive mechanical ventilation (PMV) represents a major challenge in critical care and is associated with high mortality, resource utilization, and long-term morbidity. Data from dedicated respiratory intensive care units (RICUs), particularly in resource-limited settings, remain limited. This study evaluates the clinical characteristics, management strategies, and outcomes of patients requiring PMV in a tertiary RICU.

Methods

This single-center observational case series included 40 consecutive patients requiring PMV, defined as >7 days of invasive mechanical ventilation, who were admitted to a tertiary RICU between January 2023 and March 2026. Data collected included demographics, primary diagnosis, comorbidities, APACHE II scores, microbiological profiles, ventilatory parameters, and outcomes. Standardized weaning protocols incorporating spontaneous breathing trials (SBTs), early tracheostomy, non-invasive ventilation (NIV) bridging, physiotherapy, and multidisciplinary care were applied. Successful weaning was defined as liberation from invasive ventilation without reintubation within 48 hours.

Results

The mean age was 66.5 years, with high illness severity reflected by a mean APACHE II score of 36.5. Common underlying conditions included severe pneumonia with acute respiratory distress syndrome, COPD/bronchiectasis overlap, and postoperative respiratory failure. Multidrug-resistant (MDR) Gram-negative infections, particularly *Pseudomonas aeruginosa* and *Klebsiella pneumoniae*, were frequently identified. Seventeen patients (42.5%) were successfully weaned, and 12 patients (30%) achieved decannulation. The mean duration of mechanical ventilation was 14 days, and the mean hospital stay was 48 days. Despite predicted mortality exceeding 70% in most patients, observed survival exceeded expectations. Successful outcomes were associated with early tracheostomy, culture-directed antimicrobial therapy, structured weaning protocols, early mobilization, and nutritional optimization.

Conclusions

PMV in a tertiary RICU is associated with significant morbidity but can achieve meaningful weaning success through a protocolized multidisciplinary approach. Outcomes in this high-acuity cohort compare favorably with general ICU data despite the absence of dedicated weaning units. The establishment of specialized weaning units and structured post-discharge follow-up programs may further improve outcomes in resource-constrained settings.

## Introduction

Mechanical ventilation is a cornerstone life-support intervention in critical illness, but the challenge of liberation, particularly in patients requiring prolonged ventilatory support, represents one of the most demanding aspects of intensive care medicine. The international consensus conference on weaning (Boles JM et al., (2007)) defined prolonged weaning as failure of at least three spontaneous breathing trials (SBTs) or requiring more than seven days to achieve successful extubation following the first SBT [1]. This classification is now widely adopted and broadly corresponds to Class 3 of the Weaning according to a New Definition (WIND) criteria [2].

The epidemiology of prolonged mechanical ventilation (PMV) is striking in its resource implications. Between 4% and 13% of all mechanically ventilated patients require PMV at some point [3]. This subgroup accounts for approximately 6% of ventilated patients but consumes 37% of total ICU resources and healthcare expenditure [3,4]. Reported in-hospital mortality ranges from 33% to 73%, and one-year mortality for PMV survivors exceeds 60% [1,3]. Beyond survival, the burden of post-intensive care syndrome (PICS), encompassing physical debility, cognitive impairment, and psychiatric sequelae, is profound in this population [5].

Patients requiring PMV constitute an extraordinarily heterogeneous group. Underlying conditions include severe pneumonia and acute respiratory distress syndrome (ARDS), exacerbations of chronic obstructive pulmonary disease (COPD) and bronchiectasis, neuromuscular disorders, post-surgical respiratory failure, and massive pulmonary embolism with haemodynamic compromise, among others. This heterogeneity demands individualised weaning strategies rather than a one-size-fits-all approach [6].

The burden of multidrug-resistant (MDR) organisms in respiratory ICUs further compounds the challenge of PMV. *Pseudomonas aeruginosa*, *Klebsiella pneumoniae*, *Acinetobacter baumannii*, and *Burkholderia cepacia* are among the most feared pathogens in ventilator-associated pneumonia (VAP), associated with prolonged ventilator dependence and higher mortality [7-10]. In resource-limited settings such as Indian tertiary care centres, the prevalence of MDR infections is disproportionately high, creating diagnostic and therapeutic challenges unique to this context.

Specialised weaning units (SWUs), dedicated facilities bridging the ICU and community care, have demonstrated superior weaning success rates in high-income countries [6,7]. The German WeanNet registry, encompassing over 11,000 patients, reported a weaning success rate of 64.3% in dedicated centres [7]. However, such structured units remain largely unavailable in India, necessitating that all phases of weaning, from medical management to rehabilitation to discharge planning, be delivered within the parent ICU or step-down unit [8].

In this context, we present a pilot prospective observational case series of 40 patients requiring PMV at the Respiratory ICU of Armed Forces Medical College (AFMC), Pune, a tertiary military teaching hospital serving both defence personnel and civilian referrals in western Maharashtra. To the best of our knowledge, this is the first prospective study conducted exclusively in a dedicated respiratory ICU enrolling only patients with primary respiratory diseases, a distinction that is central to the study’s research value.

The present study was undertaken with the following objectives: (1) to characterise the clinical profile, MDR infection burden, and weaning outcomes of a consecutive cohort of PMV patients managed exclusively in a dedicated respiratory ICU; (2) to describe the multidisciplinary management strategies employed, including weaning protocols, antimicrobial stewardship, nutritional support, physiotherapy, and psychosocial care; (3) to compare our outcomes with published Indian and international benchmarks; and (4) to propose a clinical framework, informed by these findings as hypothesis-generating evidence, for optimising outcomes in resource-constrained respiratory ICUs in India, with particular emphasis on the case for establishing dedicated specialised weaning infrastructure.

## Materials and methods

Study design

This was a single-centre pilot prospective observational case series with prospective two-year post-discharge follow-up, conducted at the Respiratory ICU of the Army Institute of Cardiothoracic Sciences (AICTS), a subsidiary of the Armed Forces Medical College, Pune, a tertiary care military teaching hospital. The Respiratory ICU is a dedicated 20-bed respiratory-specialised unit equipped with invasive and non-invasive ventilatory support, including BiPAP and HFNC, continuous renal replacement therapy (CRRT), haemodynamic monitoring, and 24-hour respiratory physician cover, distinguishing it from general medical or mixed ICUs. All patients admitted from January 2023 to March 2026 who required PMV (>7 days of invasive mechanical ventilation (IMV)) were eligible for inclusion.

Study participants

Data from the medical records of 40 consecutive patients requiring PMV were prospectively collected over the study period and reviewed. All eligible patients were enrolled consecutively; no exclusions were made beyond the pre-specified criterion of non-primary respiratory pathology. Patients with poly-trauma, road traffic accidents, or non-respiratory primary pathology were excluded. Data extracted included age, sex, weight, primary respiratory diagnosis, comorbidities, ASA physical status, cardiovascular status assessed by bedside echocardiography, APACHE II scores on admission, microbiological culture results with antibiotic sensitivities, ventilator parameters, weaning mode, duration of IMV, tracheostomy duration, total hospital stay, complications, including haemodynamic instability, acute kidney injury (AKI), electrolyte disturbances, pressure injuries, and psychiatric morbidity, and final outcomes.

Six patients were selected as detailed index cases to illustrate the spectrum of disease and individualised management. These were chosen by the lead investigator and senior respiratory physician to represent each of the six major diagnostic categories present in the cohort: post-surgical, bronchiectasis-COPD overlap, severe pneumonia/ARDS, pulmonary embolism, COPD exacerbation with non-invasive ventilation (NIV) failure, and obesity-related respiratory failure, ensuring clinical heterogeneity and illustrative breadth. The selection was not restricted to successful outcomes; index cases were chosen for diagnostic representativeness. Successfully weaned patients were enrolled in a structured 2-year outpatient follow-up programme at the Respiratory ICU OPD, with assessments at 3, 6, 12, and 24 months post-discharge, including pulmonary function testing, 6-minute walk distance, nutritional assessment, and psychiatric screening.

Prolonged mechanical ventilation was defined according to Boles JM et al. as failure of ≥3 SBTs or requirement of >7 days to pass an SBT [1]. Successful weaning was defined as liberation from invasive ventilatory support without the requirement for re-intubation within 48 hours. Decannulation was defined as removal of the tracheostomy tube with sustained respiratory independence. MDR organisms were defined as resistance to ≥3 antibiotic classes. VAP was diagnosed using clinical, microbiological, and radiographic criteria according to ECDC definitions.

Ethical considerations

Institutional ethical clearance was obtained prior to data collection from the Ethics Committee of the Armed Forces Medical College, Pune (IEC Approval No.: IEC/2025/760). Written informed consent was obtained from all patients or their legally authorised representatives enrolled in the follow-up programme. The study was conducted in accordance with the Declaration of Helsinki and applicable Indian Council of Medical Research (ICMR) guidelines for biomedical research. Patient data were anonymised prior to analysis, and confidentiality was maintained throughout.

Statistical analysis

Descriptive statistics were used to summarise patient demographics and clinical characteristics. Continuous variables are reported as mean ± SD or median with IQR, as appropriate, and categorical variables are reported as frequencies and percentages. The normality of continuous variables was assessed using the Shapiro-Wilk test; variables with p > 0.05 were considered normally distributed and analysed using parametric tests, while those with p ≤ 0.05 were treated as non-parametric. Between-group comparisons (weaned vs. non-weaned patients) were performed using the independent-samples t-test for normally distributed continuous variables and the Mann-Whitney U test for non-parametric data. Categorical variables were compared using the Chi-square test or Fisher’s exact test where cell counts were less than five. A p-value of <0.05 was considered statistically significant. All analyses were performed using SPSS version 26.0 (IBM Corp., Armonk, NY, USA).

Weaning protocol

A standardised daily weaning assessment was conducted by the respiratory physician and physiotherapy team. Readiness for SBT was assessed using the following criteria: resolution or partial resolution of the underlying cause of respiratory failure; FiO₂ ≤0.40 with SpO₂ ≥92%; positive end-expiratory pressure ≤8 cm H₂O; haemodynamic stability without escalating vasoactive support; and intact cough and gag reflexes. SBTs were conducted using either a T-piece or pressure support ventilation (PSV) at 5-8 cm H₂O for 30-120 minutes [9,10].

In anticipation of prolonged weaning, early elective tracheostomy was performed in patients in whom PMV was anticipated, typically those who had failed ≥3 SBTs or in whom >10-14 days of ventilatory support was expected based on clinical trajectory, underlying diagnosis, and physiological reserve. Tracheostomy was generally performed between day 7 and day 14 of mechanical ventilation, following multidisciplinary team discussion and in the absence of contraindications. This approach was undertaken to facilitate airway clearance, reduce the work of breathing, improve patient comfort, and enable early physiotherapy [8,11]. The specific timing of tracheostomy for each index case is described in the Results section.

Following tracheostomy, weaning was progressively achieved through Synchronised Intermittent Mandatory Ventilation (SIMV) reduction, transition to BiPAP via a leak valve, gradual increases in tracheostomy cuff deflation time, and ultimately T-piece trials with incrementally extended off-ventilator periods. Regarding cuff deflation in patients receiving nasogastric (NG) tube feeding, gradual tracheostomy cuff deflation was undertaken exclusively during periods when NG feeds were paused, with a minimum of 30 minutes after feed cessation, and with the patient positioned at 30-45° head elevation. Cuff deflation duration was incrementally extended in a stepwise manner based on the patient’s tolerance, swallowing assessment, and secretion burden, with early speech therapy involvement for swallowing evaluation prior to initiating oral feeding.

The early mobilisation protocol comprised a structured four-level progression: (1) passive range-of-motion exercises, commenced within 24-48 hours of haemodynamic stabilisation; (2) active-assisted limb exercises; (3) seated balance training; and (4) standing and pre-ambulation activities, delivered by the physiotherapy team twice daily and documented on a standardised physiotherapy chart. Nutritional support was initiated within 24-48 hours of ICU admission in all haemodynamically stable patients via a NG tube, with protein delivery targeted at ≥1.2-1.5 g/kg/day. Nutritional adequacy was reviewed daily based on serum albumin, pre-albumin, and tolerance of enteral feeds, with dietitian input for all patients.

## Results

The study cohort comprised 40 patients aged 58-87 years, with a mean age of 66.5 years. The cohort included 24 males (60%) and 16 females (40%); ASA physical status class was III in 28 patients (70%) and class IV in 12 patients (30%). Underlying diagnoses included bronchiectasis-COPD overlap syndrome, severe pneumonia with ARDS meeting Berlin criteria [12], massive pulmonary embolism with haemodynamic compromise, postoperative respiratory failure following thoracic surgery, obstructive sleep apnoea with structural lung disease, and morbid obesity with hypercapnic respiratory failure. All patients were admitted with primary respiratory diagnoses; patients with poly-trauma, road traffic accidents, or non-respiratory primary pathology were excluded. Most patients had at least two significant comorbidities, most commonly hypertension (HTN), coronary artery disease (CAD), type 2 diabetes mellitus (DM), and chronic kidney disease (CKD). Left ventricular ejection fraction (LVEF) data, obtained via bedside echocardiography on admission, were available for 32 of 40 patients; reduced LVEF (<50%) was documented in 9 of these 32 patients (28.1%) and may have contributed to weaning difficulty in this subgroup.

APACHE II scores at admission ranged from 31 to 43, with a mean of 36.5 ± 4.1, corresponding to predicted mortalities of 73.3-94.1%, a sobering reflection of the severity of illness in this cohort. Despite these scores, 17 patients (42.5%) were successfully weaned off IMV, substantially outperforming actuarial mortality predictions. Of these, 12 patients (30%) achieved full decannulation. The mean hospital stay was 48 days (range 30-72 days), the mean duration of ventilator support was 14 days (range 7-52 days; SD 11.2), and the mean tracheostomy duration was 42 days (range 18-95 days; SD 19.8). Weaned patients had significantly lower mean APACHE II scores compared to non-weaned patients (34.1 ± 3.6 vs. 38.4 ± 4.3; t(38) = 3.42, p = 0.001). The proportion of patients with MDR infections was significantly higher in the non-weaned group (87.0% (20/23) vs. 58.8% (10/17); χ²(1) = 4.01, p = 0.045). Length of hospital stay did not differ significantly between weaned and non-weaned patients (mean 46.3 vs. 49.2 days; Mann-Whitney U = 163.5, p = 0.48). All 17 successfully weaned patients were enrolled in a structured 2-year outpatient follow-up programme at the Respiratory OPD, AICTS, assessing pulmonary function, functional capacity, and general well-being at 3, 6, 12, and 24 months post-discharge.

MDR Gram-negative organisms were the predominant pathogens across the cohort. *Pseudomonas aeruginosa*, susceptible to colistin and/or piperacillin-tazobactam and ciprofloxacin in various isolates, was the most frequently isolated organism in tracheal and blood cultures. *Klebsiella pneumoniae*, MDR and susceptible to ceftazidime-avibactam in combination with aztreonam, was isolated in patients with severe ARDS, consistent with published data showing MDR *Klebsiella* as a leading cause of VAP-related mortality in Indian ICUs [13]. *Burkholderia cepacia*, typically a coloniser in patients with bronchiectasis, was isolated in the blood culture of one patient with BCOS and was susceptible to meropenem. *Acinetobacter baumannii* and *Serratia marcescens* were also isolated in individual cases. UTIs were caused most commonly by *Proteus* species, which were fosfomycin-susceptible.

All antibiotic therapy was guided by culture and sensitivity results, with active antimicrobial stewardship review. Novel beta-lactam/beta-lactamase inhibitor combinations, including ceftazidime-avibactam combined with aztreonam for MBL-producing strains, were deployed for carbapenem-resistant Enterobacterales (CRE) in index case 3, reflecting the growing global evidence base for this combination [14]. Adequate initial antibiotic therapy (AIAT) was established within 48-72 hours in all surviving patients. In non-surviving patients, failure to achieve AIAT was attributable to the delayed availability of microbiological susceptibility results combined with the inherent resistance profiles of MDR pathogens; failure to achieve AIAT is an independent predictor of ICU mortality in MDR *Pseudomonas pneumonia* [15]. Hypophosphataemia was identified in 11 patients (27.5%) during the ICU course and was actively corrected, given its recognised role in diaphragmatic fatigue, respiratory muscle weakness, and weaning failure. The primary weaning mode employed was SIMV reduction followed by PSV and T-piece trials; pressure-regulated volume control (PRVC) ventilation was not routinely used in this series.

Six index cases were selected to represent the range of clinical presentations and management trajectories in this cohort. Their salient features, including tracheostomy timing, are presented in Table 1.

**Table 1:** Summary of six index cases managed in the Respiratory ICU, including tracheostomy timing. ARDS: Acute respiratory distress syndrome; BCOS: Bronchiectasis-COPD overlap syndrome; CAD: Coronary artery disease; CKD: Chronic kidney disease; DM: Diabetes mellitus; HTN: Hypertension; MDR: Multidrug resistant; OSA: Obstructive sleep apnoea; PH: Pulmonary hypertension; RF: Respiratory failure; S: Susceptible; Trach.: Tracheostomy; Vent.: Ventilator; AKI: Acute kidney injury; BiPAP: Bilevel positive airway pressure; HAP: Hospital-acquired pneumonia; NIV: Non-invasive ventilation; TB: Tuberculosis.

Case	Age/Sex	Primary diagnosis	Comorbidities	Tracheostomy timing	Key organisms isolated	Hosp. days	Vent. days	Trach. days	APACHE II	Outcome
1	61/F	Postoperative diaphragmatic palsy, type II RF	DM	Day 10	*Pseudomonas* spp. (colistin-S), *Serratia* spp., *Proteus* spp.	46	29	31	31	Decannulated; discharged home
2	62/F	Bronchiectasis-COPD overlap, acute exacerbation	COPD, chronic type II RF	Day 12	*Pseudomonas* spp. (colistin-S), *Burkholderia *spp., *Acinetobacter *spp.	70	52	49	43	Decannulated; discharged home
3	69/F	Severe pneumonia with ARDS and AKI	DM, HTN, CAD, COPD	Day 9	MDR *Klebsiella* spp., *Pseudomonas* spp. treated with ceftazidime-avibactam + aztreonam	59	37	31	39	Decannulated
4	87/M	Massive pulmonary embolism, cardiac arrest, HAP	HTN, CAD, CKD	Day 11	No organism isolated	56	25	30	40	Decannulated; discharged home
5	58/M	Acute COPD exacerbation with NIV failure	HTN, CAD, COPD, PH, OSA, previous pulmonary TB	Day 14	No organism isolated	39	21	100	31	Discharged with tracheostomy and BiPAP
6	62/F	Severe pneumonia, ARDS, AKI, and metabolic acidosis	Morbid obesity	Day 8	No organism isolated	31	22	20	35	Decannulated

Across the cohort, the principal weaning modalities employed were structured SBT protocols using PSV and T-piece trials, progressive transition to BiPAP via a leak valve through the tracheostomy, and early physiotherapy-driven ambulation. In patients with COPD and chronic type II respiratory failure, such as index case 2, early NIV bridging through the tracheostomy was associated with progressive ventilator independence by resting the respiratory muscles while allowing stepwise weaning of invasive support [16,17].

Electrolyte replacement, particularly potassium and magnesium, was integral to daily management, as hypokalaemia and hypomagnesaemia impair respiratory muscle function and predispose to SBT failure [18]. CRRT was required in patients with AKI, including index cases 3 and 6, and peritoneal dialysis was instituted in the most severely affected patient, index case 3. Haemodynamic instability requiring vasoactive support was managed with noradrenaline, vasopressin, and, in one case, triple inotropic support, index case 2, with daily review and de-escalation as clinical status permitted.

Nutritional support was initiated early via NG feeding in all patients unable to maintain oral intake. Transition to oral feeding was encouraged as a rehabilitation milestone and a marker of improving functional status. In line with ESPEN ICU guidelines, protein delivery was targeted at ≥1.2-1.5 g/kg/day, recognising the accelerated lean mass catabolism associated with critical illness and prolonged ventilatory support [19,20].

Psychosocial morbidity, including depression, anxiety, and ICU-related PTSD, was identified in multiple patients [5]. Psychiatric consultation was obtained for patients identified as having clinically significant affective symptoms. A distinctive feature of our unit’s approach was the structured involvement of next-of-kin (NOK) in rehabilitation activities, including passive range-of-motion exercises, oral feeding support, communication facilitation, and psychological reassurance. This family-integrated rehabilitation model has emerging evidence supporting improved outcomes and reduced PICS burden [21].

The outcomes of the present series are contextualised against published international benchmarks in Table 2.

**Table 2:** Comparison of key outcome metrics between the present series and published international and Indian cohorts. Note: Comparative data were derived from published Indian and international cohorts, including Vora CS et al. [22], Bigatello LM et al. [23], Muzaffar SN et al. [24], and Schönhofer B et al. [7]. MV = mechanical ventilation; PMV = prolonged mechanical ventilation; SWU = specialised weaning unit.

Parameter	Present series (n = 40)	Vora CS et al. (n = 45) [22]	Bigatello LM et al. [23]	Muzaffar SN et al. (n = 49) [24]	Schönhofer B et al. (n = 11,424) [7]
Mean age (years)	66.5	32	-	-	-
Survival/weaning success (%)	42.5% weaned (17/40)	57.75% weaned/discharged; hospital mortality 42.25%	41% weaned; 6-month mortality 46%	33% ICU mortality; 65% 12-month survival	64.3% successfully weaned
Mean hospital stay (days)	48 (range 30-72)	~44	40, median	-	-
Ventilation duration	14 (range 7-52)	Mean ICU stay: 57 days	Mean MV duration: 46 days in acute care respiratory unit	>7	Median MV duration: 37 days
Mean tracheostomy days	42 (range 18-95)	-	-	-	-
OPD follow-up among survivors	100% of successfully weaned patients (17/17) enrolled in a structured 2-year OPD programme	-	-	-	-
ICU resource use	37% of ICU bed-days among PMV patients	37% of bed-days	-	37% of ICU resources	-

## Discussion

A weaning success rate of 42.5% (17/40), with full decannulation achieved in 30% (12/40), was recorded in this cohort from a dedicated Respiratory ICU. These findings should be interpreted as hypothesis-generating within the inherent constraints of a single-centre pilot observational design without a comparator group; prospective, protocol-driven, multicentre studies are needed to establish causal relationships between specific interventions and outcomes. Notwithstanding these limitations, the outcomes compare favourably with published Indian and international PMV benchmarks and are noteworthy given the exceptionally high acuity of this cohort.

In the Indian literature, Vora CS et al. (JAPI, 2015) reported on 45 patients requiring PMV (≥21 days) in a medical ICU at a tertiary care centre in Mumbai, with a hospital survival rate of 57.75%. However, this cohort had a considerably younger mean age of 32 years and included a predominance of neurological diagnoses, including acute inflammatory demyelinating polyneuropathy (AIDP), cerebrovascular accident (CVA), and tetanus. It is acknowledged that neurological diagnoses such as CVA with brainstem involvement can necessitate prolonged ventilation with significant morbidity; however, the specific profile of Vora CS et al., including younger age and predominantly peripheral neuromuscular pathology with preserved respiratory musculature once the acute illness resolves, represents a clinically distinct population from the elderly, multimorbid patients with intrinsic lung disease in our series, where pulmonary pathology itself limits weaning potential. To the best of our knowledge, this is the first study conducted exclusively in a dedicated Respiratory ICU enrolling only patients with primary respiratory diseases. Our distinct cohort represents a population with inherently severe pulmonary pathology, often presenting with advanced hypoxaemic respiratory failure and limited physiological reserve. Unlike mixed ICU settings, where outcomes may be influenced by a broader spectrum of systemic conditions, the homogeneity of our study population reflects a higher baseline disease severity, plausibly accounting for the relatively elevated mortality observed.

It is important to acknowledge that the multimorbidity burden, particularly the co-occurrence of AKI, CKD, CAD, and cardiac dysfunction alongside primary respiratory failure, represents a significant confounding influence on weaning outcomes that cannot be fully disentangled in a cohort of 40 patients without a control group. The independent contribution of each comorbidity to weaning failure cannot be established from the current data. Increasing the sample size through prospective multicentre collaboration is the critical methodological next step to permit meaningful subgroup analysis and to control for comorbidity-related confounding.

Similarly, Muzaffar SN et al. (SGPGI Lucknow, 2017) reported on 49 PMV patients with a median APACHE II score of only 17 and a 12-month survival of 65%, again in a cohort with substantially lower acuity than ours. Internationally, Bigatello LM et al. (Crit Care Med, 2007) reported on PMV patients managed in an acute care respiratory unit at Massachusetts General Hospital; while survival outcomes were reported at follow-up, the weaning outcomes should be checked carefully against the cited source before direct comparison with our 42.5% weaning success rate. The recent multicentre study by Gupta P and Lee T (CHEST, 2025) reported a 6-month mortality of 46% in PMV patients from German general ICUs without dedicated weaning infrastructure, corresponding to approximately 54% survival, compared with the 57.5% survival achieved in our Respiratory ICU series, despite our patients having far higher APACHE II scores [25]. These comparisons collectively suggest, though do not causally prove, that Respiratory ICU management with structured respiratory physician-led weaning may be associated with outcomes that are at least comparable to, or may exceed, those achievable in general ICU PMV cohorts both in India and internationally [3,4,26].

Crucially, our patients presented with exceptionally high acuity, with APACHE II scores of 31-43 predicting mortalities of 73-94%, making the 42.5% weaning success rate a clinically encouraging achievement despite high actuarial mortality risk. This outcome is particularly noteworthy given the absence of a dedicated specialised weaning unit (SWU) at our centre. ICUs with established SWUs, such as those within the German WeanNet network, have reported weaning success rates of 64.3%, highlighting that our results, while superior to those of some general ICU series, still fall short of what appears achievable with optimal step-down infrastructure. The Respiratory ICU setting itself, with specialised nursing ratios, 24-hour respiratory physician cover, dedicated physiotherapy teams, and protocol-driven weaning, may have contributed to outcomes comparable with those of general medical ICU series, but a dedicated SWU would represent the logical next step in improving outcomes further [3,7].

The German WeanNet registry data (n = 11,424) demonstrated a 64.3% weaning success rate in dedicated weaning centres, compared with 42.5% in our Respiratory ICU series [7]. The gap between our weaning success rate and that achievable in dedicated SWUs is likely attributable to a combination of factors: the absence of a step-down SWU, a higher burden of MDR infections, a higher prevalence of multimorbidity, and the inclusion of all PMV patients regardless of a priori likelihood of weaning success, a different denominator from that in most SWU studies, which pre-select candidates with recovery potential. The Bretonneau SWU experience (Almomani BA et al., 2018) similarly demonstrated that patients transferred to a dedicated respiratory weaning unit achieved 67% ventilator liberation at 90 days versus 38% for those remaining in the parent ICU, a gap closely mirroring our own series versus the WeanNet benchmark and reinforcing the argument that the infrastructure of specialised weaning care may be an independent determinant of outcome [27].

MDR Gram-negative infections were an important factor associated with outcomes in this cohort, with MDR infection prevalence significantly higher among non-weaned patients (87.0% vs. 58.8%, p = 0.045). *Pseudomonas aeruginosa* is consistently identified as one of the common MDR pathogens in respiratory ICU VAP across global registries [15,28]. Patients with MDR *Pseudomonas* pneumonia who received inadequate initial antibiotic therapy experienced significantly longer periods of mechanical ventilation after pneumonia onset compared with those who received adequate therapy, with a median of 16.5 versus 8 days in published series [15].

The isolation of *Burkholderia cepacia* in a blood culture of our BCOS patient, index case 2, deserves specific comment. *B. cepacia* complex is an inherently difficult-to-treat organism with intrinsic resistance to many antibiotics and is encountered disproportionately in patients with structural lung disease and bronchiectasis. Meropenem susceptibility, as in this case, represents a therapeutically favourable susceptibility pattern and enabled clinical cure [14].

The use of ceftazidime-avibactam in combination with aztreonam (CZA-ATM) in index case 3 with MDR *Klebsiella* and *Pseudomonas* co-infection reflects an evidence-informed approach to carbapenem-resistant Enterobacterales (CRE), particularly those harbouring metallo-beta-lactamases (MBLs). Current EUCAST and IDSA guidance supports CZA-ATM for MBL-producing CRE when aztreonam retains activity [14,29]. The successful decannulation of this patient following CRE infection underlines the life-saving potential of novel antibiotic combinations when deployed in a culture-guided, stewardship-governed framework.

Early elective tracheostomy was associated with several clinical benefits in our cohort, including improved airway clearance, reduced work of breathing, facilitation of patient communication, and enablement of early physiotherapy. A multicentre Japanese cohort (Tanaka A et al., 2022) demonstrated that hospital mortality was significantly associated with tracheostomy timing in a stepwise fashion, with early tracheostomy (≤6 days) associated with the lowest mortality [11]. The ISCCM expert panel recommendations for tracheostomy in Indian ICUs (Swami V et al., 2020) support the use of early tracheostomy in patients with anticipated prolonged mechanical ventilation (>10-14 days), noting that early tracheostomy is associated with increased ventilator-free days without significantly impacting VAP incidence [8]. Our practice of performing elective tracheostomy in patients with anticipated PMV aligns with these recommendations and is reflected in the mean tracheostomy-to-decannulation interval of 42 days in our cohort. It should be noted, however, that in the absence of a control group, we cannot establish whether early tracheostomy independently caused improved outcomes or whether it was a marker of patients with greater recovery potential who would have done well regardless [30].

ICU-acquired weakness (ICUAW), affecting up to 25% of mechanically ventilated patients, is a major driver of weaning failure, prolonged hospital stay, and post-discharge disability [31]. All surviving patients in our cohort received physiotherapy-driven early ambulation programmes, including passive and active range-of-motion exercises, supported sitting, tilt-table training, and bedside walking as clinical status permitted. A 2023 systematic review and meta-analysis by Wang L et al., encompassing multiple RCTs, demonstrated that systematic early mobilisation was associated with significantly reduced ICU length of stay (MD -2.18 days, 95% CI -4.22 to -0.13, p = 0.04) and duration of mechanical ventilation (MD -2.27 days, 95% CI -3.99 to -0.56, p = 0.009) in mechanically ventilated adult ICU patients [32]. A 2021 Cochrane-format meta-analysis by Menges D et al. confirmed improvements in muscle strength and physical function with systematic early mobilisation [33].

A key feature that distinguished our approach was the early transition to BiPAP support via the tracheostomy using a leak valve, which allowed progressive nocturnal ventilatory rest with daytime weaning trials. This technique is particularly valuable in patients with chronic hypercapnic respiratory failure, including COPD, BCOS, and OSA overlap, where respiratory muscle fatigue must be carefully managed and home BiPAP is ultimately the planned long-term support strategy [16,17,34].

Malnutrition is both a cause and a consequence of prolonged ventilatory dependence. The catabolic state of critical illness accelerates muscle protein breakdown at a rate that standard nutritional formulations may not fully counteract, particularly when full caloric delivery is delayed by haemodynamic instability or gastrointestinal intolerance [19]. ICU-acquired muscle wasting has been quantified ultrasonographically as a reduction in quadriceps muscle layer thickness over the ICU stay, underscoring the severity and speed of lean mass loss in this population [20]. In our practice, early enteral nutrition via a nasogastric tube was commenced within 24-48 hours of ICU admission in all haemodynamically stable patients. Oral feeding was introduced as a rehabilitation milestone, both as a functional measure and as a psychosocial motivator. Serum albumin levels were monitored as an adjunctive nutritional/prognostic marker, recognising that hypoalbuminaemia is independently associated with weaning failure [3,35].

Depression, anxiety, and post-traumatic stress disorder (PTSD) are highly prevalent in survivors of prolonged critical illness [5]. Jackson JC et al. (2014) demonstrated in the BRAIN-ICU cohort that 37% of survivors of critical illness met diagnostic criteria for PTSD, 33% had anxiety, and 26% had depression at three-month follow-up [5,36]. Active psychiatric consultation was sought for patients demonstrating significant affective symptoms, as untreated depression may impair motivation for weaning participation and rehabilitation adherence.

Our structured incorporation of next-of-kin as active participants in rehabilitation, supporting physiotherapy exercises, facilitating oral feeding, providing psychological reassurance, and participating in tracheostomy cuff closure training, reflects a family-integrated ICU care model. This approach aligns with the PICS-Family (PICS-F) framework and the SCCM’s ABCDEF bundle: Assess, prevent, and manage pain; Both spontaneous awakening and breathing trials; Choice of analgesia and sedation; Delirium monitoring; Early mobility; and Family engagement and empowerment [21,34].

Lone and Walsh (2011) estimated that establishing a dedicated three-bed regional weaning unit for a population of 1.2 million could save approximately £344,000 annually while improving weaning outcomes [3]. The German SWU experience (WeanNet) documents that over 60% of patients deemed “unweanable” in the ICU achieve successful weaning in a dedicated centre [7]. Dolinay T et al. (2024) further confirm that more than 50% of patients considered ventilator-dependent in the ICU will achieve liberation in post-acute care settings [26]. The contrast with non-SWU, non-respiratory-specialised settings is instructive: Bigatello LM et al. (2007) reported that only 41% of PMV patients managed in an acute care respiratory unit without dedicated weaning infrastructure were successfully weaned, and Ponfick et al. (CHEST, 2025) observed a 6-month mortality of 46% in PMV patients from general ICUs without SWUs in Germany. In India, Vora CS et al. and Muzaffar SN et al. both documented hospital mortality exceeding 33-42% in PMV cohorts managed in general ICUs without dedicated respiratory physician weaning teams. In contrast, dedicated SWUs with full multidisciplinary teams achieve 60-64.3% liberation rates, as documented by WeanNet Germany. The gap between our 42.5% weaning rate and the SWU benchmark of 64.3% is quantifiable, and the establishment of a formal step-down weaning unit represents a logical next step, though it is acknowledged that external benchmarks, rather than internal controlled data, underpin this recommendation [26].

Our cohort, managed entirely within a specialised Respiratory ICU without a dedicated step-down SWU, achieved a 42.5% weaning success rate against predicted mortalities exceeding 80% in most patients. A dedicated SWU with multidisciplinary staffing, including an intensivist, respiratory physician, physiotherapist, speech therapist, dietitian, psychiatrist, and trained rehabilitation nurses, would be expected to improve upon these outcomes, as suggested by international SWU registries. The 2-year structured follow-up programme initiated for all successfully weaned patients in this cohort has enabled longitudinal tracking of pulmonary function recovery, functional capacity via 6-minute walk distance, and psychological well-being; these data will be reported prospectively as a separate study.

Limitations

This study carries inherent limitations that must be explicitly acknowledged. First, the single-centre design and overall cohort of 40 patients limit generalisability, and the study is best characterised as a pilot prospective observational case series. Second, although the study design is prospective observational, a component of data extraction relied on retrospective medical record review for historical variables, introducing the possibility of incomplete data capture and documentation bias; the post-discharge follow-up component is fully prospective and protocol-driven. Third, the absence of a comparison group managed without the specific weaning strategies described makes causal attribution of outcomes to individual interventions impossible; all associations reported should be interpreted as hypothesis-generating rather than causally definitive. Fourth, the selection of six illustrative index cases for detailed narrative may introduce an element of positive presentation bias; readers should note that 23 of 40 patients (57.5%) did not achieve successful weaning. Fifth, the multimorbidity burden, including AKI, CKD, CAD, and cardiac dysfunction, represents a confounding influence on weaning outcomes that cannot be temporally controlled or disentangled in this cohort. Sixth, systematic echocardiographic data on ejection fraction were available only in a subset of patients (32/40), limiting conclusions about the independent contribution of cardiac dysfunction to weaning outcomes. Seventh, some variables, including formal PICS assessment at follow-up, detailed spirometry, and 6-minute walk distances post-discharge in all patients, were not uniformly available for all patients at every time point. Prospective, protocol-driven data collection and regional multicentre collaboration would substantially strengthen future analyses from this setting.

## Conclusions

In this pilot prospective observational case series of 40 high-acuity patients (APACHE II scores 31-43) requiring PMV in a dedicated Respiratory ICU, a weaning success rate of 42.5% (17/40) and full decannulation in 30% (12/40) were achieved with protocolised, multidisciplinary care, despite high actuarial mortality predictions. To the best of our knowledge, this is the first study conducted exclusively in a dedicated Respiratory ICU enrolling only patients with primary respiratory diseases. Successful weaning was associated with culture-directed MDR infection management, early elective tracheostomy, structured SBT and progressive BiPAP bridging protocols, physiotherapy-led early mobilisation, nutritional optimisation, and psychosocial and family-integrated support. These findings are hypothesis-generating; causal relationships between specific interventions and outcomes require prospective multicentre validation. All weaned patients were enrolled in a structured 2-year post-discharge follow-up programme; this prospective dataset will be reported separately. Future research should focus on larger multicentre cohorts, prospective evaluation of PRVC versus SIMV-based weaning strategies, and the impact of dedicated specialised weaning units in the Indian healthcare context, with the establishment of such a unit representing the most impactful next step to close the gap between our 42.5% weaning rate and the 64.3% achieved in international dedicated weaning centres.
